# Supplementary material for: An Emerging Visible-Light Organic–Inorganic Hybrid Perovskite for Photocatalytic Applications
Source: Nanomaterials (Basel). 2020 Jan 7;10(1):115. doi: 10.3390/nano10010115 (PMC7023354; doi:10.3390/nano10010115)
Supplement: Supplementary file 1 [file nanomaterials-10-00115-s001.pdf]

Supporting information

An Emerging Visible-Light Organic–Inorganic Hybrid Perovskite for Photocatalytic Applications

Bianca-Maria Bresolin [1,*], Samia Ben Hammouda [1] and Mika Sillanpää [1,2]

[1] Laboratory of Green Chemistry, School of Engineering Science, Lappeenranta University of Technology, Sammonkatu 12, 50130 Mikkeli, Finland; samiabenhammouda@gmail.com (S.B.H.); Mika.Sillanpaa@lut.fi (M.S.)

[2] Department of Civil and Environmental Engineering, Florida International University, Miami, FL 33174, USA.

* Correspondence: Biancabresolin@yahoo; Bianca-maria.Bresolin@lut.fi

Nowadays, the energy demand is dramatically increased due to the remarkable growing of world population and industrial activities, this implies important environmental risks. Thus, sustainable supplies and clean energy sources are widely considered key factors to develop green technology and preserve life on our planet. Solar energy, due to its availability, accessibility, and cleanliness could address in the same time such urgent environmental and energy issues. In particular, visible light as major portion of solar spectrum, non-toxic and renewable source of energy seems to be a sustainable way to address the as mentioned problems. Among of the available sources for visible light irradiation halogen was selected in the current study (Visilight CL150). The main features of the as employed device are summarized in **TableS1**.

Table S1: Main characteristics of the employed halogen lamp

Lamp	Power consumption (W)	Luminous efficacy (lumens/watt)	Colour temperature (K)	Average Lifespan (hours)
**Visilight CL150**	150	16-24	3270	1.000

Morover, **Figure S1** illustrates the active spectrum of a generic halogen light. In particular, halogen lamps usually show a region of higher homogeneous intensity between 550 and 650 nm.

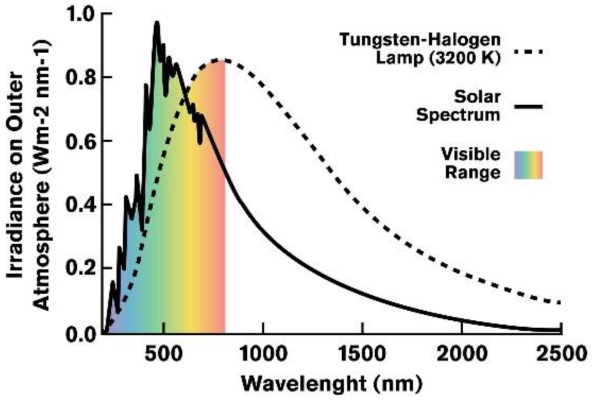

Figure S 1*:* Spectra comparison between a Halogen lamp (3200K) and the solar irradiance on a surface per unit wavelength with emphasis on visible light range

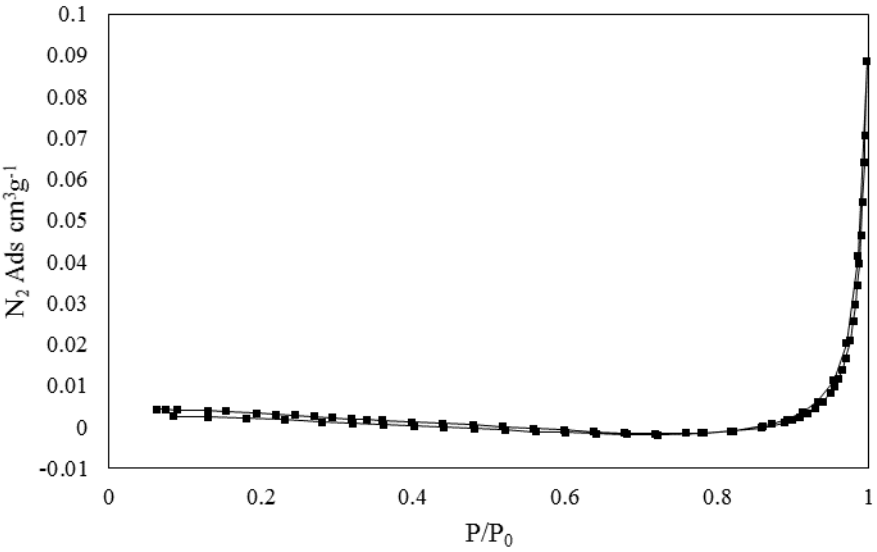

Figure S 2: N_2 sorption isotherms

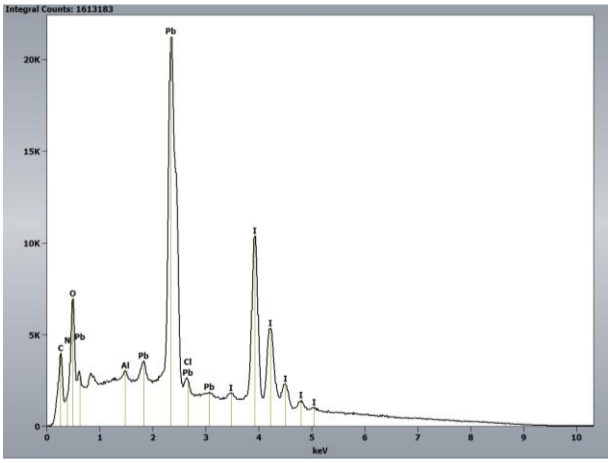

Figure S 3: EDS spectra of the as prepared $CH_3NH_3PbI_3$ catalyst

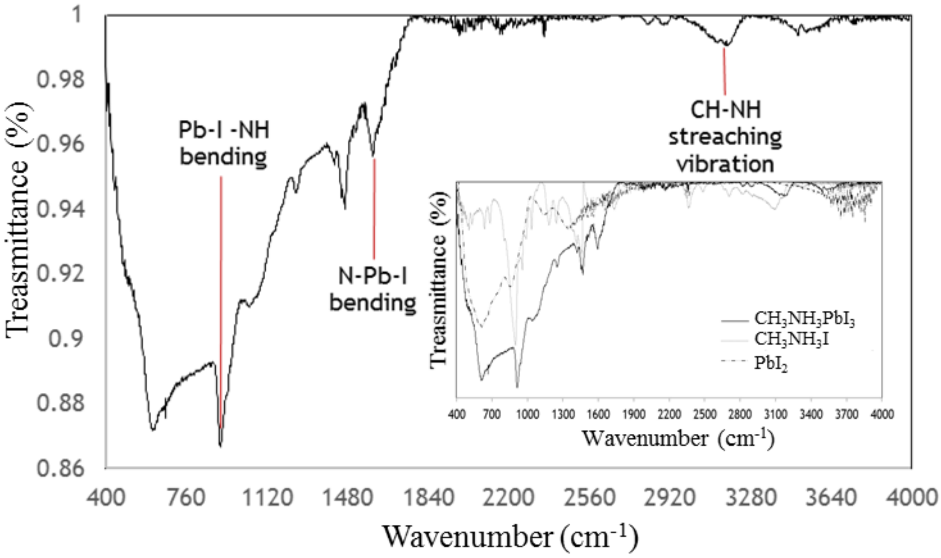

Figure S 4: FT-IR spectrum of the as prepared $CH_3NH_3PbI_3$ catalyst. Comparison between final powder and precursors (small window)

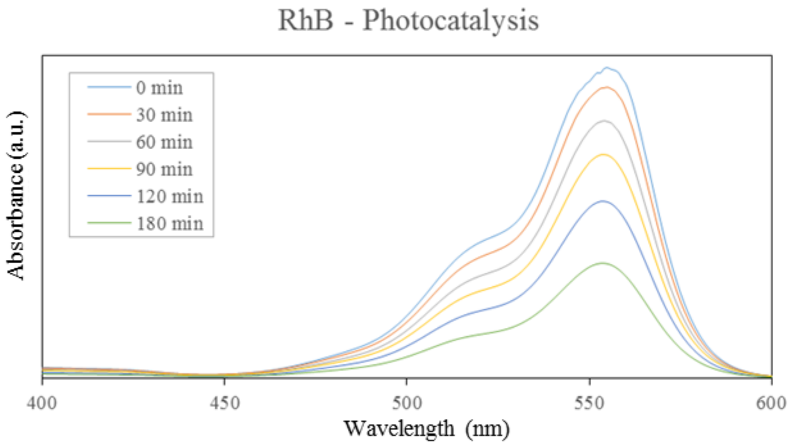

Figure S 5: RhB temporal peak extinction.

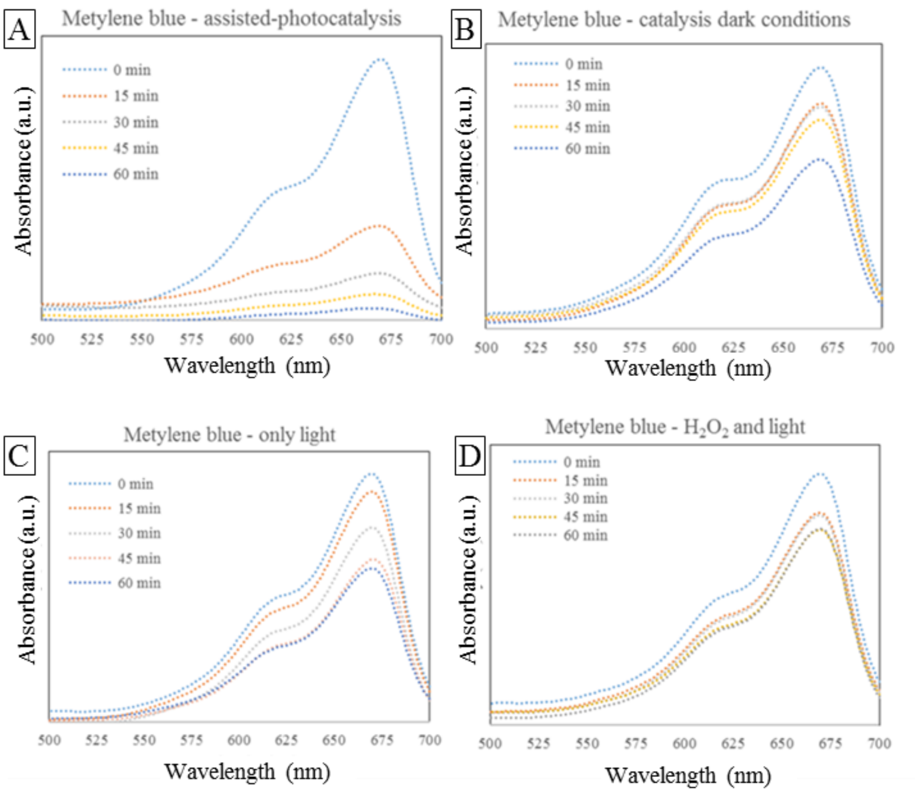

Figure S 6: Methylene blue temporal peak extinction performed under different conditions. A) $CH_3NH_3PbI_3$ assisted-photocatalytic process under visible light, B) $CH_3NH_3PbI_3$ catalytic process in dark conditions, C) MB treatment under light irradiation, D) MB treatment under light and peroxide oxidant assistance. In all cases, the former conditions were used: 10^{-4} M H_2O_2, 10 mg/L MB, halogen lamp, 0.5 g/L catalyst load.
